# Supplementary material for: Hybrid SARS-CoV-2 immunity among frontline workers in a high-tourism setting: A community-based serosurvey in Ambergris Caye, Belize, June 2022
Source: PLOS Glob Public Health. 2026 May 11;6(5):e0006204. doi: 10.1371/journal.pgph.0006204 (PMC13160308; doi:10.1371/journal.pgph.0006204)
Supplement: Attachment — Submitted filename: Review Report for Manuscript PGPH.pdf [file pgph.0006204.s001.pdf]

**Review Report for Manuscript PGPH-D-25-02807**

**"Hybrid SARS-CoV-2 Immunity Among Frontline Workers in a High-Tourism Setting: A Community-Based Serosurvey in Ambergris Caye, Belize, June 2022"**

**Overall Considerations**

**General Strengths:**

- **Clarity and Organization:** The manuscript is exceptionally clear and well-organized across all its sections, facilitating ease of reading and comprehension of the findings and arguments.
- **Robust Alignment:** There is a very strong and consistent alignment among the study objectives, the employed methodological strategy, data analysis, and the presented conclusions.
- **Comprehensive Statistical Analysis:** The statistical approach is sophisticated and appropriate for the data, employing methods that enable the identification of independent factors.
- **Ethical Transparency:** The clear and specific mention of ethical approvals demonstrates a commitment to responsible research practices.
- **Critical and Contextualized Discussion:** The Discussion section stands out as a strong point, integrating results critically with the literature, proposing hypotheses for unexpected findings, and connecting them to practical implications.
- **Transparent Reporting of Limitations:** The honesty and depth in describing the study's limitations contribute significantly to the work's credibility.
- **Public Health Relevance:** The study addresses a highly relevant public health issue in tourism-dependent contexts, providing actionable data.

**General Weaknesses:**

- The only general, and relatively minor, weakness observed pertains to the formal description of the psychometric properties of the qualitative data collection instrument (the questionnaire).

**General Suggestions for Improvement:**

- Review and expand the description of the questionnaire used, focusing on its psychometric qualities or justifying their absence, as detailed in the specific suggestions for the Methodology section.
- Ensure that supplementary figures, though not included in this review version, are impeccably formatted, clear, and intuitive in the final version.

**Section-by-Section Considerations**

**1. Methodological Strategy and Method Description**

**Adequacy to Objectives: ADEQUATE**

- **Analysis:** The methodological strategy employed is **ADEQUATE** for the study's objectives, which include: (1) measuring SARS-CoV-2 antibody seroprevalence, (2) assessing COVID-19 exposure history and COVID-19-like illness symptoms, and (3) determining infection risk factors. The community-based seroepidemiological approach, utilizing questionnaires and anti-spike and anti-nucleocapsid serological tests, is directly aligned with these objectives.

**Method Description: ADEQUATE**

- **Analysis:** The method description is notably detailed and clear.
  - **Subjects/Participants:** The description is excellent, detailing the target population (hotel/restaurant employees in the tourism sector and government workers in Ambergris Caye), eligibility criteria (age, residency, employment type), sample size calculation, and recruitment process (random hotel selection, refusals, efforts to achieve participant numbers, inclusion of various worker types).
    - **Examples:**
      - *Materials and Methods, Study Population and Recruitment*: "Our target population in Ambergris Caye was: (1) hotel/restaurant employees in the tourism sector, and (2) government workers, including first responders (police and fire), teachers, and Town Council staff."
      - *Materials and Methods, Eligibility Criteria*: "Eligible individuals were (1) current government or tourism employees; (2) 18 years of age or older; (3), living in Ambergris Caye for at least three months; and (4) planned to continue living in the community for at least another six months."
  - **Data Collection Procedure:** This is very well detailed, specifying the use of a "standardized survey" for demographic information, medical history, COVID-19 vaccination history, and epidemiological exposures. It mentions data collection on paper forms and entry into REDCap, as well as language support. For blood samples, it describes the venipuncture process, processing within two hours, aliquotting, storage, and transport. The ELISA kits used (InBios SCoV-2 Detect IgG ELISA kit and Bio-Rad Platelia SARS-CoV-2 Total Ab Assay kit) are named, along with their targets (spike and nucleocapsid protein) and purpose, including their respective sensitivity and specificity.
    - **Examples:**
      - *Materials and Methods, Data Collection*: "A standardized survey was used to collect demographic information, medical history, COVID-19 vaccination history, and epidemiological exposures."
      - *Materials and Methods, Laboratory Procedures*: "Serum samples were screened for the presence of SARS-CoV-2 specific IgG and total Ig antibodies using commercial ELISA kits... We

used the InBios SCoV-2 Detect IgG ELISA kit... with a reported sensitivity and specificity of 100%... We also used the Bio-Rad Platelia SARS-CoV-2 Total Ab Assay kit... with a reported sensitivity and specificity of 98% and 99.3%, respectively."

- **Data Analysis:** The statistical analysis is well-described, mentioning the software tools (Stata V18, NCSS 2025, GraphPad) and types of analysis: descriptive statistics, simple logistic regression, and multivariable logistic regression (backward stepwise approach, p<0.25 for inclusion, and p<0.05 for retention criteria).
  - **Examples:**
    - *Materials and Methods, Statistical Analyses*: "All statistical calculations were performed in Stata V18... Descriptive statistics were used to calculate proportions. Simple logistic regression was used to identify risk factors... Multivariable logistic regression models were constructed to identify independent factors associated with nucleocapsid positivity."

**Psychometric Qualities of Instruments/Scales: REQUIRES REVISION (for the questionnaire)**

- **Analysis:** For **laboratory assays**, yes, performance qualities (sensitivity and specificity) are provided. However, for the **questionnaire** used, there is no explicit mention of its psychometric qualities (construct validity, reliability, etc.), which constitutes a gap.
- **Suggestions for Improvement:**
  - Even if the questionnaire was developed specifically for the study, it would be beneficial to briefly mention the process of its development, for instance, whether it was reviewed by experts for clarity and comprehensiveness (content validity).
  - If any part of the questionnaire was adapted from existing instruments, even if not a formal "scale," reference to the original source and the adaptation process would be valuable.

**Original Title of Scales/Instruments and Reference Source for Validation/Adaptation: ADEQUATE (for assays); REQUIRES REVISION (for the questionnaire)**

- **Analysis:** For **laboratory assays**, yes, the manuscript provides the full commercial names of the ELISA kits (InBios SCoV-2 Detect IgG ELISA kit and Bio-Rad Platelia SARS-CoV-2 Total Ab Assay kit) and manufacturers, as well as citing references for product information (References 9 and 10). For the **questionnaire**, it is referred to as a "standardized survey" and based on the WHO UNITY protocol, but there is no original title or specific validation/adaptation reference source for the questionnaire itself.
- **Suggestions for Improvement:**
  - Clarify whether the "standardized survey" is a modified version of the WHO UNITY protocol or an original tool. If it's an adaptation, briefly describe the adaptation process. If it's an original tool, indicate that it was developed for the study.

- Consider adding the reference source for the WHO UNITY protocol directly in the discussion of the questionnaire to more clearly link the methodological basis.

---

**2. Data Analysis Articulated with Objectives and Theoretical Framework**

**Articulation: ADEQUATE**

- **Analysis:** The data analysis is **excellently articulated** with the study's objectives and underlying theoretical framework.
  - **Articulation with Objectives:**
    - **Measure seroprevalence:** The methodology with anti-spike and anti-nucleocapsid IgG tests, and the results with seropositivity proportions (99% anti-spike, 81% anti-nucleocapsid), are direct and appropriate.
    - **Assess exposure/symptom history:** Questionnaires collected the information, and descriptive statistics (Tables 1 and 2) and simple logistic regression address this objective.
    - **Determine risk factors:** Multivariable logistic regression, described and applied, identifies independent factors associated with nucleocapsid positivity (Table 3).
  - **Articulation with Theoretical Framework:**
    - **Hybrid Immunity:** The study is grounded in the concept of hybrid immunity, differentiating antibodies to understand protection.
    - **Epidemiological Risk Factors:** The investigation of factors such as household size, contact, masks, and vaccination aligns with infectious disease epidemiology principles. Multivariable logistic regression is the standard tool for this.
    - **Vulnerability in Specific Contexts:** The choice of the population in Ambergris Caye reflects the theory that specific occupations and environments increase risk.
    - **Antibody Waning:** The discussion of nucleocapsid antibody seroreversion demonstrates an understanding of the theoretical aspects of immune response kinetics.

---

**3. Ethical Aspects**

**Ethical Consideration and Committee Approval: ADEQUATE**

- **Analysis:** Ethical aspects were duly considered, and research ethics committee approval was clearly mentioned.
  - **Example:** In the *Materials and Methods* section, under the subheading "Study Population and Recruitment," the text explicitly states: "This protocol was

approved by the BCM Institutional Review Board (H-50470) and the Belize MOHW."

  - The mention of the protocol number (H-50470) adds traceability.

---

**4. Main Results**

**Clarity and Objectivity:** ADEQUATE

- **Analysis:** The main results are presented **very clearly and objectively** in the Results section.
  - **Descriptive Subheadings:** The section is well-structured with clear subheadings (*"Overall participant population"*, *"SARS-CoV-2 spike protein seropositivity and vaccination history"*, etc.), guiding the reader.
  - **Numbers and Percentages:** Results are quantified with exact numbers and percentages, facilitating comprehension.
    - **Examples:**
      - "Of 551 participants, 428 (78%) were tourism workers and 123 (22%) government employees."
      - "almost all (n=547; 99%) were seropositive for IgG against the SARS-CoV-2 spike protein."
      - "A high proportion of participants (440/551, 80%) were nucleocapsid positive..."
  - **Use of Tables and Figures:** Table 1, Table 2, and Table 3 are effective in presenting data in an organized manner, highlighting Odds Ratios and p-values.
  - **Highlighting Associations:** Statistically significant associations are explicitly mentioned with aOR and p-values.
    - **Examples:**
      - "nucleocapsid positivity was independently associated with having >5 people in household (aOR=2.08, p=0.018), while receiving a COVID-19 booster was protective (aOR=0.57, p=0.013)."
      - "Living with ≥5 people in the household significantly increased the odds of nucleocapsid positivity (OR=2.15, p=0.01)."
  - **Objectivity:** The presentation is factual, reserving discussions and interpretations for the appropriate section.

---

**5. Comparison Between Research Results, Data, and Literature Concepts**

**Construction of Comparison:** ADEQUATE

- **Analysis:** The comparison in the Discussion section is **very well constructed**, interlinking the findings with existing scientific knowledge.
  - **Direct Connection and Theoretical Support:** The discussion links high seroprevalence to "hybrid immunity," citing studies (Refs. 22 and 23) that reinforce the idea of enhanced protection.
    - **Examples:**
      - *Discussion*: "High seroprevalence of both vaccine- and infection-induced antibodies was observed, highlighting the emergence of hybrid immunity among frontline workers."
      - *Discussion*: "Prior research has found hybrid immunity influenced by both vaccination and natural infection synergistically heightens the level and duration of protection [22, 23]."
  - **Corroboration with Literature:** The finding of large households as a risk factor is corroborated with similar studies in Andorra, the Dominican Republic, and Nicaragua (Refs. 14, 15, 16).
    - **Examples:**
      - *Discussion*: "We found that participants with ≥5 people in the household were at higher risk of infection. This is consistent with findings from a study based in the microstate of Andorra... Another study in the Dominican Republic in 2021 found that risk of being seropositive... A population-based seroprevalence study in Nicaragua demonstrated increased seropositivity..."
  - **Critical Discussion of Unexpected Findings:** The surprising finding regarding hand sanitizer use is addressed critically, with plausible hypotheses and references to WHO and CDC guidelines (Refs. 17, 18).
    - **Examples:**
      - *Discussion*: "A surprising result was that participants who always/usually carried hand sanitizer were more likely to be seropositive for nucleocapsid antibodies. The WHO and Centers for Disease Control and Prevention (CDC) both recommend frequent handwashing... We hypothesize that carrying hand sanitizer may be an indication of the individuals' higher risk perception... An alternative hypothesis is that carrying hand sanitizer can provide a false sense of security... Additionally, carrying hand sanitizer might not necessarily correlate with usage."
  - **Methodological Validation and Theoretical Limitations:** The validity of using anti-nucleocapsid antibodies is discussed, but also the limitations of seroreversion (Refs. 19, 20), illustrated with data from the study itself.
    - **Examples:**

        - *Discussion*: "These findings support the validity of using assays that detect antibodies against the nucleocapsid antigen... One limitation of this approach is that total Ig antibodies to the nucleocapsid protein wane over time... In our study, 20 participants who tested negative for nucleocapsid antibodies reported both a history of a positive COVID-19 test..."
    - **Public Health Implications:** Results are consistently contextualized with their practical implications for public health interventions.

---

**6. Conclusions or Final Considerations**

**Clarity and Support by Results:** ADEQUATE

- **Analysis:** The conclusions and final considerations are **clear and solidly supported by the study's results**.
    - **Study Context:** The conclusion reaffirms the study's importance in the high-risk population within a tourism-dependent setting.
        - **Example:** *Conclusion*: "This study provides a unique opportunity to assess vaccination coverage and risk factors for COVID-19 infection among a high-risk population..."
    - **Key Results:** It highlights high vaccination uptake (98%) and anti-spike antibody prevalence (99%), and hybrid immunity due to high anti-nucleocapsid antibody prevalence (81%), all direct results.
        - **Example:** *Conclusion*: "Vaccination uptake was high as evidenced by self-report and 99% prevalence of anti-SARS-CoV-2 spike protein antibodies. The high seroprevalence for nucleocapsid protein indicates that most of the study population has hybrid immunity through both infection and vaccination."
    - **Relevance and Implications:** Emphasizes its relevance as the first study in the Central American tourism industry and its capacity to inform future public health interventions.

---

**7. Contributions and Limitations**

**Description of Contributions and Limitations:** ADEQUATE

- **Analysis:** The study's main contributions and limitations are **clearly described** in the Discussion and Conclusion sections.
    - **Contributions:**
        - **Unique Context:** Study in a high-risk population in a high-tourism area.
        - **Evidence of Hybrid Immunity:** Demonstrates the occurrence of hybrid immunity.

        - **Pioneering:** First study focused on Central American tourism.
        - **Informing Public Health:** Helps to inform future interventions.
    - **Limitations (explicitly in the Discussion section):**
        - **Sampling Bias:** Difficulty in having a complete sampling frame.
            - **Example:** *Discussion*: "First, it was challenging to have a complete sampling frame, making the participant selection subject to bias."
        - **Recall and Response Bias:** Risk of bias in collecting clinical and behavioral history.
            - **Example:** *Discussion*: "There is a risk for recall and response bias. Participants might have felt influenced to respond positively to questions related to behaviors to prevent infections, including wearing masks and carrying hand sanitizer. This could contribute to our finding that carrying hand sanitizer was associated with nucleocapsid positivity."
        - **Exclusion of Vaccine Types/Uncertainty:** Small number of participants with inactivated virus vaccines and uncertainty about vaccine type in some cases, potentially influencing nucleocapsid analysis.
            - **Example:** *Discussion*: "Another limitation worth noting is that we had a small number (n=6) of participants who reported vaccination with Beijing Sinopharm's BBIP-CorV inactivated whole virus vaccine... it is possible some of these 29 participants received Sinopharm... Nevertheless it could have influenced the statistical analysis."

---

**8. References**

**Pertinence and Sufficiency: ADEQUATE**

- **Analysis:** The 24 references included are **highly pertinent and sufficient** for the manuscript's scope.
    - **Pertinence:** They cover the geographical and socioeconomic context of Belize, methodology (WHO UNITY protocol, ELISA kits), comparative discussion with literature (seroprevalence studies, immunological concepts like antibody kinetics and hybrid immunity), and public health implications (WHO/CDC guidelines).
    - **Sufficiency:** The quantity is appropriate, and the breadth is robust.
        - **Timeliness:** Most references are recent (2020-2025), crucial for the COVID-19 topic.

        - **Diversity of Sources:** Includes high-impact journals, health organization websites, government data, and vaccine trackers, indicating comprehensive and reliable research.
        - **Comprehensive Support:** All key statements and discussions are adequately supported by references.

---

**9. Supplementary Files**

**Information, Structure, and Necessity:** ADEQUATE

- **Analysis:** The manuscript refers to three crucial supplementary figures which, though not directly appended to the review version, are **essential** for the clarity and completeness of the work.
    - **Figure 1 (Testing algorithm):** Likely a flowchart, crucial for understanding the laboratory methodology and inferences about immunity. **Necessary.**
    - **Figure 2 (Study population and sample for analysis):** A flowchart showing the recruitment process, exclusions, and final sample, fundamental for transparency and assessing representativeness. **Necessary.**
    - **Figure 3 (Timing of first COVID-19 vaccination vs. reported cases):** A comparative graph of participant vaccination timing against the epidemiological curve in Belize, providing crucial context for interpreting results. **Necessary.**
    - **Structure:** The suggested structures (flowchart, time-series graphs) are the most appropriate for the information they intend to present.
