# Supplementary material for: Hybrid SARS-CoV-2 immunity among frontline workers in a high-tourism setting: A community-based serosurvey in Ambergris Caye, Belize, June 2022
Source: PLOS Glob Public Health. 2026 May 11;6(5):e0006204. doi: 10.1371/journal.pgph.0006204 (PMC13160308; doi:10.1371/journal.pgph.0006204)
Supplement: Attachment — Submitted filename: Response to reviewers.docx [file pgph.0006204.s003.docx]

*We greatly appreciate the kind and thoughtful review of our manuscript. We incorporated the valuable feedback and feel the paper is now strengthened as a result. Please find our responses to the concerns raised:*

Journal Requirements:

1. Please provide additional details regarding participant consent. In the ethics statement in the Methods and online submission information, please ensure that you have specified (1) whether consent was informed and (2) what type you obtained (for instance, written or verbal, and if verbal, how it was documented and witnessed). If your study included minors, state whether you obtained consent from parents or guardians. If the need for consent was waived by the ethics committee, please include this information.

 Response: This is now included in the Ethics statement section of the methods.

If you are reporting a retrospective study of medical records or archived samples, please ensure that you have discussed whether all data were fully anonymized before you accessed them and/or whether the IRB or ethics committee waived the requirement for informed consent. If patients provided informed written consent to have data from their medical records used in research, please include this information.

 Response: Not applicable.

2. Please include a complete copy of PLOS’ questionnaire on inclusivity in global research in your revised manuscript. Our policy for research in this area aims to improve transparency in the reporting of research performed outside of researchers’ own country or community. The policy applies to researchers who have travelled to a different country to conduct research, research with Indigenous populations or their lands, and research on cultural artefacts. The questionnaire can also be requested at the journal’s discretion for any other submissions, even if these conditions are not met. Please find more information on the policy and a link to download a blank copy of the questionnaire here: https://journals.plos.org/globalpublichealth/s/best-practices-in-research-reporting. Please upload a completed version of your questionnaire as Supporting Information when you resubmit your manuscript.

Response: This is now included in the supporting information section of the resubmission.

3. Please send a completed 'Competing Interests' statement, including any COIs declared by your co-authors. If you have no competing interests to declare, please state "The authors have declared that no competing interests exist".

 Response: This is now included on the title page.

4. Please amend your detailed Financial Disclosure statement. This is published with the article. It must therefore be completed in full sentences and contain the exact wording you wish to be published.

   a. State the initials, alongside each funding source, of each author to receive each grant. For example: "This work was supported by the National Institutes of Health (####### to AM; ###### to CJ) and the National Science Foundation (###### to AM)."

b. State what role the funders took in the study. If the funders had no role in your study, please state: “The funders had no role in study design, data collection and analysis, decision to publish, or preparation of the manuscript.”

 Response: This is now included.

5. In the online submission form, you indicated that “
   Deidentified data are available upon request, contingent upon approval of a data sharing agreement.”

   All PLOS journals now require all data underlying the findings described in their manuscript to be freely available to other researchers, either
   1. In a public repository,
   2. Within the manuscript itself, or
   3. Uploaded as supplementary information.

   This policy applies to all data except where public deposition would breach compliance with the protocol approved by your research ethics board. If your data cannot be made publicly available for ethical or legal reasons (e.g., public availability would compromise patient privacy), please explain your reasons by return email and your exemption request will be escalated to the editor for approval. Your exemption request will be handled independently and will not hold up the peer review process, but will need to be resolved should your manuscript be accepted for publication. One of the Editorial team will then be in touch if there are any issues.

Response: We have uploaded the dataset with personal identifying information removed (i.e., date of birth) and have placed it in a public repository (Harvard Dataverse). This is also now referenced in the manuscript in the methods. We also uploaded to the public repository the questionnaire used for data collection.

6. Please provide separate figure files in .tif or .eps format.

   For more information about figure files please see our guidelines:

   https://journals.plos.org/globalpublichealth/s/figures
   https://journals.plos.org/globalpublichealth/s/figures#loc-file-requirements

Response: Completed.

7. If the reviewer comments include a recommendation to cite specific previously published works, please review and evaluate these publications to determine whether they are relevant and should be cited. There is no requirement to cite these works unless the editor has indicated otherwise. 

Please review your reference list to ensure that it is complete and correct. If you have cited papers that have been retracted, please include the rationale for doing so in the manuscript text, or remove these references and replace them with relevant current references. Any changes to the reference list should be mentioned in the rebuttal letter that accompanies your revised manuscript. If you need to cite a retracted article, indicate the article’s retracted status in the References list and also include a citation and full reference for the retraction notice.

Response: Completed.

Reviewer #1: **Recommendation: Extensive Revisions**

While the manuscript is methodologically sound, ethically rigorous, and well-written, the critical issue of **data availability** necessitates **extensive revisions**. The current statement regarding data availability ("available upon request, contingent upon approval of a data sharing agreement") does not comply with PLOS Global Public Health’s policy for unrestricted data sharing. The authors must either:
1. Deposit all de-identified raw data in a publicly accessible, recognized repository and provide the corresponding access link (DOI) in the manuscript.
2. Provide a compelling justification for any restrictions that fully aligns with the "rare exception" clause of the PLOS data policy, along with a transparent mechanism for data access that minimizes barriers.

Response: Data has now been uploaded into a public repository (Harvard Dataverse) and referenced in the manuscript.

In addition to this primary concern, I recommend addressing the following minor points to further enhance the manuscript's quality during revision:
* **Questionnaire Psychometric Properties:** In the "Data Collection" section, although the "standardized survey" is mentioned, there is no discussion of its psychometric properties (e.g., how content validity was ensured, if it was piloted, or if it's an adaptation of a validated tool). A brief clarification on the development or adaptation process of the questionnaire would strengthen the methodological description.
* **Clarity on "Standardized Survey" Source:** While it mentions being based on the WHO UNITY protocol, explicitly state whether the survey was directly adopted, adapted, or newly developed for this study, providing a reference for the original if applicable.

Response: This information is all now detailed in the methods section, and we added the survey tool used in the study to the public repository.

Reviewer #2: The manuscript describes a well-executed study with strong laboratory methods and addressing a relevant and interesting public health topic. However, certain required elements are unclear and revision is recommended to improve the clarity and transparency of the study.

The Introduction does not adequately explain why studying hybrid immunity is important in the current context. Since this is central theme of the paper, a clearer rationale would strengthen the background. Similarly, the choice of tourism workers as the study population needs a but more justification. Although the objectives are stated, they do not consistently align with the results.

Response: Thank you for this helpful feedback. This is now added.

The study is clearly a cross-sectional study, but it is not stated in the methodology or even the title or the abstract. The Sampling approach seems a bit complex. The description of the cluster sampling can be explained more clearly considering some selection bias. The exact recruitment period and duration of the data collection is also not mentioned anywhere. Sample Size is unclear, especially with different denominators across variables. While statistical analysis is strong, the manuscript should mention how missing data were handled.

Response: Changes made.

The descriptive data given in tables and also neatly explained in the results section, but each table has a lot of variables, makes it slightly difficult to interpret. The prevalence of the nucleocapsid positivity which is the natural infection is reported also the anti spike IgG ELISA positivity is reported but the hybrid immunity is not interpreted.

Response: Tables were moved to the end of the document and placed into landscape to make it easier to see the variables for interpretation. Changes made.

The Discussion section contains valid points but not well organized and doesn't relate with study title and objectives and lacks clear narrative on hybrid immunity which is the theme of the study.

Response: Changes made.

The conclusion does not fully answer all objectives and fails to emphasize on the implications for public health and tourism-sector risk.

Response: Changes made.

A uniform style of referencing to be followed throughout, the text should use superscript numerical citations as per Vancouver style of referencing.

Response: We followed examples from published articles in PLoS Global Public Health, and the references were in brackets with normal font size.

Overall, This is a good study with strong technical work and it would benefit from clearer reporting. The study needs to align with STROBE guidelines to improve clarity, and build coherence between objectives, methods, results and conclusions.

Response: Thank you!

Reviewer #3: The research is relevant to PLOS Global Public Health and it was well written. However, I could not see the statement on the data availability/accessibility for the study. In addition, the abstract should have been structured as it is better for presentation and readership. Conflict of interest was not declared in the study.

Response: These are now included. For the abstract, the journal guidelines state it should have no headings and be unstructured. We have made sure the background, methods, results, and conclusions flow better.
